# Decoding the divide: what distinguishes apoptosis-induced proliferation from compensatory proliferation?

**DOI:** 10.1186/s12964-025-02336-3

**Published:** 2025-07-10

**Authors:** Andreas Bergmann, Yun Fan

**Affiliations:** 1 Department of Molecular, Cell and Cancer Biology, UMass Chan Medical School, 364 Plantation Street, Worcester, MA 01605 USA; 2 School of Biosciences, University of Birmingham, Birmingham, B15 2TT UK

Compensatory Proliferation (CP) and Apoptosis-induced Proliferation (AiP) are related processes that promote cellular proliferation in response to tissue loss, mechanical injury, ionizing radiation, or chemical damage. While CP and AiP share similarities, such as their roles in maintaining tissue homeostasis and facilitating repair, they are defined by distinct differences that have frequently led to confusion and occasional conflation within scientific discourse. Another source of confusion is that “undead” cells—immortalized cells with activated apoptotic signaling but blocked execution—are classified under AiP, even though they do not undergo apoptosis. Therefore, the current definitions fall short of adequately clarifying and differentiating CP from AiP [11]. Here, we aim to clarify the distinctions between CP and AiP by examining their definitions, initiating signals, and the specific contexts in which they contribute to tissue repair and homeostasis.

CP describes the process whereby surviving cells detect tissue damage or loss, and proliferate to restore normal organ size and function (Fig. 1, left panel). Importantly, CP can be initiated through multiple distinct mechanisms: it can be triggered by non-apoptotic forms of cell death, mechanical cues resulting from disruption of tissue architecture, or even systemic factors that sense overall reduction of tissue mass. In fact, CP can occur entirely independently of cell death signals, as surviving cells may respond directly to changes in tissue mechanics, altered cell–cell contact or other cues. This versatility highlights CP as a robust regenerative system that ensures tissue homeostasis through multiple redundant pathways. The unifying principle of CP is that remaining healthy cells ultimately respond by proliferating in a controlled manner to replace lost tissue mass, regardless of the initial stimulus [2].

**Fig. 1:**
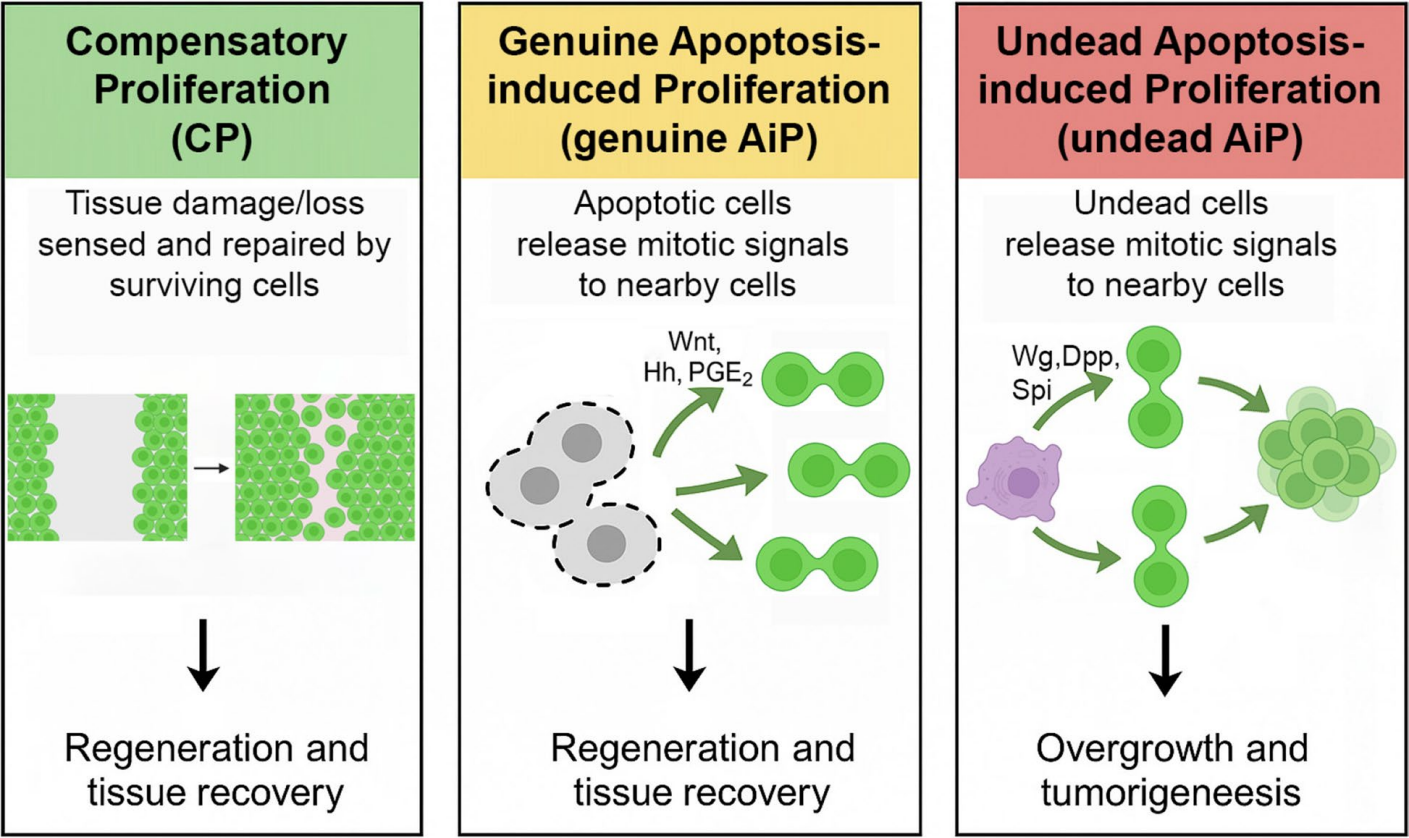
Schematic illustration of CP and AiP (genuine and undead). Wg, Dpp and Spi are *Drosophila* homologs of Wnt, BMP and EGF, respectively. Dividing cells are illustrated by  . For details, see text. Created with Biorender

Examples of CP are found in systems like the liver, where partial hepatectomy leads to the proliferation of remaining hepatocytes to restore tissue mass without significant apoptosis [5, 19]. Similarly, in *Drosophila,* after X-ray-induced damage to imaginal discs, surviving cells undergo proliferation to ensure that the tissue grows to its normal size, even if a significant portion of the cells are lost [1, 8]. This form of proliferation and regeneration is regulated mainly through growth factors and pathways such as the JAK/STAT and Hippo pathways, ensuring tissue size is restored to normal.

AiP, on the other hand, is a specialized form of CP that occurs when apoptotic cells – instead of undergoing passive clearance – actively stimulate mitosis in nearby surviving cells (Fig. 1, middle panel) [6, 12]. AiP ensures that tissues continue to develop or regenerate even when a significant proportion of cells undergo apoptosis. A defining feature of AiP is the involvement of apoptotic caspases, which not only execute cell death, but also contribute to AiP by actively releasing growth-promoting signals like Wnt, Hedgehog (Hh) or Prostaglandin E2 (PGE2) which trigger nearby cells to proliferate (Fig. 1, middle panel) [2, 3, 7, 10, 13, 14, 17]. This dual role of caspases highlights the paradoxical nature of apoptosis, where death signals can contribute to life by promoting AiP and regeneration [2].

AiP has been extensively studied in *Drosophila* imaginal discs, where activation of apoptotic caspases such as the initiator caspase Dronc triggers mitogenic signaling from apoptotic cells [9, 13, 17]. There are two types of AiP models in *Drosophila*: "genuine" AiP, where apoptotic cells complete their death process, but still release mitogenic signals (Fig. 1, middle panel) [3, 4, 18], and "undead" models, where apoptotic cells are kept immortalized by blocking effector caspase activity (Fig. 1, right panel) [4, 7, 14]. Although technically not undergoing apoptosis in its terminal form, undead cells can still secrete mitogenic signals [6] which can trigger excessive overgrowth rather than a balanced tissue recovery (Fig. 1, right panel). As such, undead cells rely on apoptotic signaling to produce mitogenic factors, which is why they are categorized under AiP. This is different from CP, which does not rely on apoptotic signaling, but instead uses surviving cells to directly promote tissue growth in a cell autonomous manner.

AiP has also significant implications in pathological conditions, particularly cancer. For example, after treatments like irradiation, apoptotic tumor cells can release the AiP signal PGE2 that stimulates proliferation of surviving tumor cells, potentially contributing to tumor regrowth [10]. This mechanism could substantially impact cancer therapy strategies. Furthermore, tumor cells can exhibit properties similar to "undead" cells in AiP [16] in which apoptotic signals that are intended to induce cell death, can paradoxically promote further cell growth, contributing to tumor progression and resistance to therapy [7, 15]. This phenomenon creates a complex dynamic that challenges traditional understanding of cancer cell behavior.

In summary, while both CP and AiP involve cellular proliferation to maintain or restore tissue integrity, the fundamental difference lies in the origin of signaling. CP is controlled by autonomous signals from surviving cells responding to tissue loss, whereas AiP is driven by apoptotic and undead cells that send signals to promote the proliferation of nearby cells. This distinction is important because the two processes are often confused. CP relates primarily to recovery from tissue damage, while AiP highlights the signaling role of apoptotic cells, which can have broader biological consequences, including tissue overgrowth and tumor development. These distinct mechanisms demonstrate how organisms balance tissue loss and regeneration, and how cancer cells exploit apoptotic pathways for growth.

## Data Availability

No datasets were generated or analysed during the current study.
